# Reclaiming or rebranding marketing: implications beyond digital

**DOI:** 10.1007/s13162-020-00178-5

**Published:** 2020-10-26

**Authors:** Thomas Ritter

**Affiliations:** Department of Strategy and Innovation, Copenhagen Business School, Frederiksberg, Denmark

## The domain of marketing

Ted Moser’s interesting views on the changing role of marketing raise a fundamental question of timeless interest: What is marketing? Ted offers three central observations. First, we need to move beyond the division between “digital” and “analogue” to think about marketing as a whole, of which some parts are digital, and others are not. Second, Ted argues that marketing is taking territory from sales, which implies that marketing does not restrict itself to general communication, market-offering presentations, and lead generation. It often completes the buying cycle and, thereby, generates sales. Third, marketing is undergoing a necessary transition. While I recognize and agree with these arguments based on my own work, we need to ask whether it is, or was, meaningful to divide marketing into different parts and assign them different labels. In other words, we need to think about what marketing is.

There is a distinction between a functional view of marketing and a capability-based view of marketing, which is also known as the distinction between “Marketing as a function (big M) in relation to marketing as a process … (little m)” (Marketing Science Institute Research Priorities 1996, p. 6, in Moorman and Rust 1999, p. 180). While the former is an organizational design issue (i.e., what activities should be placed in the organizational unit called “Marketing”), the latter relates to the substance of marketing—the activities and processes that constitute marketing. As an organizational capability, marketing’s position in firms has grown stronger, driven by consumer individualism, social media connectedness, and the availability of data. As a function (i.e., a job and department label), marketing is facing a life-threatening implosion—marketing departments are becoming smaller and moving further away from the top of the organization. When former marketing activities are allocated to other departments, their label changes too. In other words, small m is exploding, while big M is imploding. If this trend continues, the term “marketing” will be devalued and eventually disappear, at least from the strategic landscape of organizations. Alternatively, this trend could be reversed and marketing could take back important activities, potentially supported by digitalization, as pointed out by Ted.

While there is no universally agreed, timeless definition of the marketing domain, I prefer to divide the domain into four complementary processes (Ritter and Andersen 2010): market learning and market interacting (based on market sensing and market linking as proposed by Day 1994) plus market prioritizing (e.g., Homburg et al. 2008; Wetzel et al. 2014) and market shaping (e.g., Mele et al. 2015; Storbacka and Nenonen 2011).

Market learning includes segmentation, competitor analysis, and customer-journey mapping—all of the organizational processes that build on, extend, and update an organization’s knowledge about its markets. Organizations use market insights to make decisions about customers, offerings, communication, and innovation. As organizations are restricted in their resource base and as different paths relate to different performance outcomes, market prioritization, that comprises deciding on what to do—and what not to do—is an essential activity. Market interacting covers such areas as market communication, branding, relationship management, omni-channel marketing, and customer-journey management—all processes that bring customers and organizations in contact with each other. Finally, market shaping acknowledges the fact that markets are neither given nor stable. Markets change in terms of their rules, standards, and practices. This area has been popularized by the notion of creating “blue ocean strategies” (Kim and Mauborgne 2004) and it has expanded into the development of capabilities for market shaping (e.g., Nenonen et al. 2019).

As we subscribe to a capability perspective (as, e.g., debated in this journal by Jaworski and Lurie 2019, and Morgan 2019), the definition of marketing must be based on processes that produce outcomes. Moreover, the measure of marketing capability must capture how well and how persistently an organization is able to perform the processes based on its employees’ skills and qualifications as well as resource inputs (e.g., Winter 2003). Interestingly, such processes are often allocated to different individuals, teams, and units in organizations, which results in coordination costs and complications. Subsequently, high levels of marketing capability can only be achieved when all processes are well coordinated with each other.

**Fig. 1:**
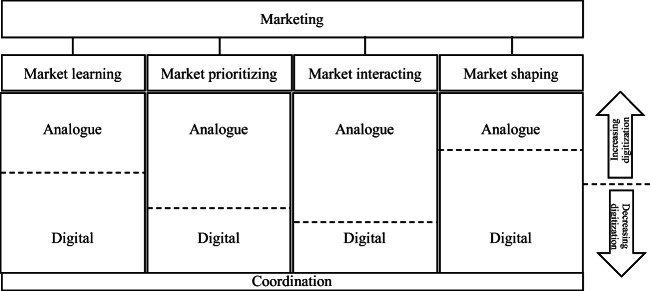
Marketing, four marketing processes, and levels of digitization and coordination

While I personally prefer “marketing” as the overarching term (as illustrated in Fig. 1), its application as an umbrella term going forward requires a re-interpretation, such that the various sub-processes (e.g., sales, market analytics, consumer insights) are encompassed under the marketing umbrella. The reality in many firms is very different, as the sales department is often the owner of customer relationships and responsible for turnover, while marketing is typically viewed as a sales-support function. Just as Ted highlights several examples of new job titles and departments, I have noted that firms and consultants alike adopt new terms. Marketing is now often referred to as “commercial excellence,” and the new layer above sales and marketing is the “chief commercial officer.” Thus, instead of re-interpreting marketing and reclaiming the original territory, marketing has been re-branded as commercialization. Is this the best way forward? Should marketing reclaim its true, inclusive meaning? It is hard to imagine the academic journals rebranding themselves (e.g., the *Journal of the Academy of Commercialization Science* and the *Journal of Commercialization*). It is also hard to allow the continued use of different terms by academia and practitioners, which serves to increase the gap between them.

This leaves academics with great research opportunities. First, the development of a consistent conceptual framework of processes and associated capabilities should offer a mapping of the relevant field which is free of conceptual ambiguity. The development of a common ground will enable comparison, replication and accumulation of research results and thus serve as solid foundation for the field—also enabling the development of relevant performance measures and discussions of antecedents. For this grand endeavor, all available research methods from conceptual to empirical, from qualitative to quantitative, from primary to secondary data are suitable to guide our field out of the confusion and into the light of well-defined and commonly accepted concepts.

Second, I call for a theory-in-use study (Zeithaml et al. 2020) of the domain called marketing, sales and commercialization to develop an understanding of what practitioners think about the field and the different terms. If academics develop a theory-for-theory in clear contrast to executives’ theory-in-use, we lose even more relevance despite any rigor our work may have. Maybe the term commercialization “signals to the marketplace a more wholistic, encompassing of the end to end go-to market strategy”, as Bernhard Jaworski put it in our discussions for this article.

## The digitization of marketing

Digitization (i.e., the increasing availability of data in digital formats) and subsequent digitalization (i.e., the use of digital data to invent and transform business models) have profound impacts on marketing, customer relationships, markets, and buyers (e.g., Ritter and Pedersen 2020). Given the four marketing processes discussed above, I can mirror Ted’s arguments: censor technologies, smart phones, imaging, etc., produce massive amounts of data on customers and vendors related to products, services, needs, and behaviors. This requires new market-learning capabilities and offers new ways of segmenting markets. Data also provides new bases and tools for decision making, and new ways of getting priorities right. In addition, digitization creates new means of interacting. For instance, as a result of the COVID-19 crisis, many customer contact points were moved to online formats, such as video meetings, YouTube presentations, and virtual-reality solutions. Finally, data establishes new market standards and new rules for competing.

In a nutshell, data, digitization, and digitalization affect and transform all marketing processes, but they do not make analogue fully redundant. As Ted points out, digital marketing is one part of marketing. We need to understand marketing’s digital value proposition in digital forms of value demonstrations as well as analogue value propositions and analogue value demonstrations, and their combinations. These elements cannot be separated but must be holistically understood and managed. Figure 1 illustrates the four marketing processes described above and the fact that digitization happens in all four processes but not necessarily to the same extent. Therefore, the level of digitalization may well be different (for illustration purposes indicated by the different heights of the dotted line). The speed of digitalization also may differ. By mapping an organization’s marketing capability along processes and digitization levels, and by adding the owners of the processes and their organizational positions and units, we create a starting point for developing marketing capabilities.

In line with Ted’s argument that marketing reclaims parts of sales through online channels, digital and digitization can reunite parts of marketing that have long been separated and viewed as different cultures (Homburg and Jensen 2007). While different cultures may exist (just like researchers and practitioners suggest a difference between product and service business units; Ulaga and Loveland 2014), success can be fostered by combining and aligning the different processes and their owners, not by dividing marketing into atoms.

However, digitalization creates new dilemmas. Not only do customers expect firms to use insights from apps, sensors, and devices to offer better value propositions, but they also expect vendors to respect their privacy. To this end, firms increasingly face the challenge of “doing right,” as standards and norms develop quickly and, at times, unpredictably. In other papers, we have highlighted the importance of including permission capability as an important dimension of a firm’s digitalization capability (Ritter and Pedersen 2020)—no organization can benefit from digital if it is unlawful, antagonizes its ecosystem partners, or violates societal norms and trends. Therefore, digitalization offers new insights, new revenue streams, new business models, and transformation possibilities (e.g., marketing and sales development) but only on the basis of investments in capabilities.

The research opportunities of digitization for our field are far-reaching. Beyond describing and defining the new digital elements and forms of, e.g., market offerings and market communications, research has already addressed itself to strategic choices between and combinations of digital and analogue parts in different areas of marketing. Yet, this research is only the beginning beginning of the development of a deep understanding of digitalization. But digitization may also challenge existing marketing theories—as digital forms offer new ways of testing theories, is there any “old” theory of stimulus-response or communication effectiveness which digital data and analytics can challenge? Academics can challenge established paradigms with new insights—and reinforce established wisdom with better methods (Pedersen and Ritter 2020). As such, digitization of marketing can have a fundamental impact on marketing theory.

Digitization is also a great invitation for more cross-disciplinary research: I hope for a wave of great studies that are designed and executed in teams with marketing and information technology faculty, or teams of marketing and ethics colleagues to develop the ethical digital marketing and digital ethics in marketing. This development will also challenge university administration: does a top-level publication in a different field suggest promotion in marketing? As such, digital offers challenges for practice and for academia beyond the immediate digital marketing sphere.

## The alignment of marketing

The move toward a holistic view and alignment within the marketing area (or the “commercialization domain”) does not stop by moving the boundary between sales and marketing. As Ted points out, digital has a ripple effect on marketing—and marketing has to align with the rest of the organization, including new business models. There is also a “loop ripple effect,” as marketing offers the organization new inputs, such as information on the need to differentiate and individualize offerings. The increase in the availability and use of data more closely connects marketing to other organizational functions. For instance, delivery and service units have direct access to customer data and add customer data to the system, and real-time information about customer-usage patterns provides the basis for better planning of production and service schedules. As marketing becomes more data driven and more accountable for results, it also becomes closer, better aligned, and more integrated with other parts of organizations. Digitization offers improved ways of achieving new heights in market orientation, which refers to the “organization-wide generation of market intelligence, dissemination of intelligence across departments, and organization-wide responsiveness to it” (Jaworski and Kohli 1993, p. 53). In summary, digitization can not only unite marketing but also better align marketing with the organization, help position marketing more centrally, and enable a higher market orientation. This is a truly unique chance for the marketing profession.

Research has for a long-time analyzed opportunities and challenges around the collaboration of marketing with finance, production, R&D, logistics, and others (e.g., Ellinger 2000; Gupta et al. 1986; Hyman and Mathur 2005). The new flow of digital data offers an opportunity to revisit these areas to reveal if, and potentially how, digitization can change communication and collaboration across units and departments. Digitization does not only change an individual’s workflow, or a team’s collaboration, or the interaction with customers—it also influences the way organizations and their parts can align and co-create value. Again, data not only enables change but also uniquely documents such changes, so that researchers can document and analyze such changes and their implications. These new opportunities can supplement traditional ways of analysis such as case studies and surveys.

## Marketing going forward

Ted proposes a set of transitions that marketing must undergo and enable in the wider context. I support the idea that marketing has to change in order to understand and develop new tools, methods, and technologies in order to build new capabilities for driving corporate performance. Marketing also has to reinvent and reposition itself in organizations and academia alike. Thus, beyond the capability challenge, there are challenges related to the mindset and our fundamental understanding. Not only do we need to find a way to deal with “digital”—we also need to find out what we mean by “marketing.”

## References

[CR1] Day G (1994). The capabilities of market-driven organizations. Journal of Marketing.

[CR2] Ellinger AE (2000). Improving marketing/logistics cross-functional collaboration in the supply chain. Industrial Marketing Management.

[CR3] Gupta AK, Raj SP, Wilemon D (1986). A model for studying R&D–marketing interface in the product innovation process. Journal of Marketing.

[CR4] Homburg C, Droll M, Totzek D (2008). Customer prioritization: Does it pay off, and how should it be implemented?. Journal of Marketing.

[CR5] Homburg C, Jensen O (2007). The thought worlds of marketing and sales: Which differences make a difference?. Journal of Marketing.

[CR6] Hyman MR, Mathur I (2005). Retrospective and prospective views on the marketing/finance interface. Journal of the Academy of Marketing Science.

[CR7] Jaworski BJ, Kohli AK (1993). Market orientation: Antecedents and consequences. Journal of Marketing.

[CR8] Jaworski BJ, Lurie RS (2019). Building marketing capabilities: Principles from the field. AMS Review.

[CR9] Kim WC, Mauborgne R (2004). Blue ocean strategy. Harvard Business Review.

[CR10] Mele C, Pels J, Storbacka K (2015). A holistic market conceptualization. Journal of the Academy of Marketing Science.

[CR11] Moorman C, Rust RT (1999). The role of marketing. Journal of Marketing.

[CR12] Morgan NA (2019). Researching marketing capabilities: Reflections from academia. AMS Review.

[CR13] Nenonen S, Storbacka K, Windahl C (2019). Capabilities for market-shaping: Triggering and facilitating increased value creation. Journal of the Academy of Marketing Science.

[CR14] Pedersen, C. L. & Ritter, T. (2020). What kind of scientist are you? NatureIndex.com, 13 November 2019, https://www.natureindex.com/news-blog/what-kind-of-scientist-are-you.

[CR15] Ritter T, Andersen H (2010). Building the foundation of a firm’s market competence. Marketing Review St. Gallen.

[CR16] Ritter T, Pedersen CL (2020). Digitization capability and the digitalization of business models in business-to-business firms: Past, present, and future. Industrial Marketing Management.

[CR17] Storbacka K, Nenonen S (2011). Scripting markets: From value propositions to market propositions. Industrial Marketing Management.

[CR18] Ulaga W, Loveland JM (2014). Transitioning from product to service-led growth in manufacturing firms: Emergent challenges in selecting and managing the industrial sales force. Industrial Marketing Management.

[CR19] Wetzel HA, Hammerschmidt M, Zablah AR (2014). Gratitude versus entitlement: A dual process model of the profitability implications of customer prioritization. Journal of Marketing.

[CR20] Winter SG (2003). Understanding dynamic capabilities. Strategic Management Journal.

[CR21] Zeithaml VA, Jaworski BJ, Kohli AK, Tuli KR, Ulaga W, Zaltman G (2020). A theories-in-use approach to building marketing theory. Journal of Marketing.

